# From Selection to Use: Aptamers as Targeting Reagents in Hematology

**DOI:** 10.3390/biomedicines14030534

**Published:** 2026-02-27

**Authors:** Brandon Albert, Fiona Ebanks, Kimia Gharagozloo, Xinying Hai, Raymond Ngu, Sietse Munting, Maureen McKeague

**Affiliations:** 1 Department of Chemistry, McGill University, 801 Sherbrooke Street West, Montréal, QC H3A 0B8, Canada; brandon.albert@mail.mcgill.ca (B.A.); fiona.ebanks@mail.mcgill.ca (F.E.); sietse.munting@mail.mcgill.ca (S.M.); 2 Department of Pharmacology and Therapeutics, McGill University, 3655 Promenade Sir-William-Osler, Montréal, QC H3G 1Y6, Canada; kimia.gharagozloo@mail.mcgill.ca (K.G.); xinying.hai@mail.mcgill.ca (X.H.); raymond.ngu@mail.mcgill.ca (R.N.)

**Keywords:** aptamers, hematopoiesis, SELEX, myeloid cells, lymphoid cells, cell sorting, drug delivery, therapeutics, aptasensors

## Abstract

Aptamers are synthetic nucleic acid ligands that have been proposed as alternatives to antibodies for targeting molecules and cells. In hematology, most reviews have organized aptamer literature around diseases or technological platforms. This framing has obscured how unevenly different blood cell types have been covered. In this review, we present developed aptamers organized by blood cell lineages. Specifically, we examine aptamers for B cells, T cells, natural killer cells, and red blood cells. This organization revealed a strong concentration on a small set of canonical surface markers and on malignant cell models. A parallel gap appeared in aptamers that distinguish differentiation stages or functional cell states. Within this framework, we evaluated reported applications, design strategies, and experimental use cases alongside persistent limitations in target selection and biological resolution. Our analysis highlighted both practical constraints and conceptual blind spots in current blood-cell-targeting aptamer research. Together, these observations defined a set of clear opportunities for expanding aptamer development toward more state-resolved, biologically informative, and clinically relevant targeting strategies.

## 1. Introduction

Blood is composed of multiple circulating cell populations, with its proper function depending on maintaining the correct cellular composition and functional states [1]. Blood simultaneously supports oxygen transport, immunity, and hemostasis. As such, the relevant cell types must be reliably identified and distinguished, and in some contexts, selectively targeted. This cellular diversity is not incidental; it is generated by tightly regulated hematopoiesis in the bone marrow, which produces lineages that differ in maturity, abundance, and activation state [2,3]. As a result, even within a single lineage, clinically meaningful phenotypes can reflect shifts in differentiation stage or cell state, rather than the appearance of an entirely new cell type. Disruption of these regulated programs alters both cell identity and function and can manifest as hematologic disease [4,5]. Together, the diversity and dynamic nature of blood cell populations create fundamental challenges for both diagnostic detection and therapeutic targeting.

Blood disorders and diseases describe conditions that interfere with specific proteins, receptors, and/or cellular signaling pathways [6,7]. These conditions can potentially disrupt the production, maturation, and function of blood elements. From a diagnostic and therapeutic perspective, many blood disorders are defined not only by genetic or molecular alterations, but also by changes in cell identity or surface marker expression. To address such conditions, a variety of strategies targeting blood cells and their microenvironment have been developed. These include small molecule inhibitors, stem cell transplantations and antibody therapies [8,9,10]. Each of the listed technologies face their own challenges, resulting in several unmet needs within the domain of blood diagnostic and therapeutics. This includes less toxic drugs, faster and less demanding diagnostic strategies, and difficulty in identifying and developing sensors for suitable surface targets [11,12,13].

Aptamers have been introduced as a unique tool to address key obstacles and unmet needs in blood diagnostics and therapeutics. Aptamers are short, single-stranded nucleic acids with defined tertiary structures that can be selected to bind molecular or cellular targets. These molecules have been explored for their ability to be highly specific and stable with reduced off-target effects while functioning in complex biological environments [14,15]. This review will highlight the potential of aptamers to fill existing gaps in blood diagnostics and therapeutics by showcasing their advantages and applications in this area. The review will cover relevant publications and will close with a discussion of major challenges and future perspectives for aptamer-based diagnostics and therapeutics in the context of blood and blood cell diseases.

### 1.1. Hematopoiesis

**Figure 1:**
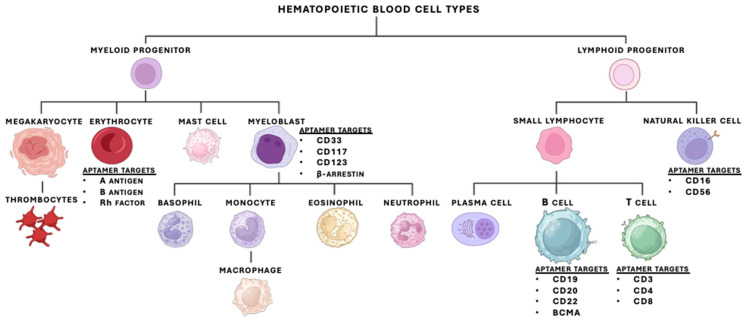
Hematopoietic differentiation and current coverage of blood cell targets by aptamers. Schematic of hematopoietic differentiation from hematopoietic stem cells (HSCs) through myeloid and lymphoid progenitor lineages to major circulating blood cell types. Blood cell types and differentiation stages for which aptamers have been reported are annotated with representative surface markers. The figure highlights both the distribution of existing aptamer targets across hematopoietic lineages and the gaps in coverage, emphasizing cell types and developmental stages that remain underrepresented in the literature. Created in BioRender. McKeague, M. (2026) https://BioRender.com/3czy3a9.

Hematopoiesis is a vital process that originates from hematopoietic stem cells (HSCs) in the bone marrow. Through tightly regulated differentiation programs, this process generates a wide range of blood cell populations with distinct lineage identities and functional states. As blood cells progress through differentiation, they acquire lineage- and state-specific phenotypes that are reflected in their surface marker expression. For example, leukocytes, which include monocytes, granulocytes and lymphocytes, defend the body against infection and injuries through innate and adaptive immune responses. On the other hand, erythrocytes and platelets represent additional terminally differentiated lineages with specialized biological roles (e.g., oxygen. CO_2_, and nutrient transport) [16]. All these populations arise through hierarchical differentiation pathways that generate phenotypically related but molecularly distinct cell types (Figure 1). Notably, blood cells also express surface markers during differentiation, several of which have been identified. Consequently, this diversity in antigen presentation amongst different hematopoietic cells offers unique targets for diagnostics and drug development. As such, molecular targeting strategies that can exploit differences in surface marker expression, including aptamer-based approaches, are well positioned to address challenges in blood cell-specific detection and intervention.

### 1.2. Blood Diseases

Blood diseases, or hematologic disorders, impact blood or blood-forming tissues, disrupting the balance needed for healthy blood production and function [17,18]. These pathologies target specific proteins, receptors, and cellular factors. This often results in abnormal cell identity, differentiation, or abundance within the blood compartment. For example, acute leukemias, such as Acute Myeloid Leukemia (AML) and Acute Lymphoblastic Leukemia (ALL), cause a rapid and uncontrolled proliferation of immature blood cells that overwhelm the bone marrow and crowd out healthy cells [19,20]. In contrast, chronic leukemias such as Chronic Myeloid Leukemia (CML) and Chronic Lymphocytic Leukemia (CLL) progress more slowly and alter immune cell composition over time [21,22]. Other blood cancers, including Hodgkin’s and non-Hodgkin’s lymphomas, arise from lymphocytes and spread through lymphatic tissues, while multiple myeloma affects plasma cells and compromises immune function through malignant bone marrow infiltration [23,24]. In addition to malignancies, inherited non-cancerous disorders such as sickle cell disease alter red blood cell morphology, reducing oxygen transport and increasing the risk of vascular occlusion [25]. Despite their diverse etiologies and clinical courses, these conditions share a common feature, namely disruption of the blood’s ability to perform its life-sustaining roles.

The biological and clinical diversity of blood disorders highlights the need for molecular tools that distinguish diseased from healthy blood cells, as well as closely related cell types and functional states within a lineage. In practice, many current treatment strategies rely on targets that are not fully specific, leading to substantial off-target effects and reduced quality of life for patients. As a result, therapeutic intervention often trades efficacy for toxicity, particularly in diseases where malignant and healthy cells share surface features. The same challenge arises in diagnostics, where the accurate identification of rare or phenotypically similar cell populations is essential for early detection and disease monitoring. Therefore, there is a need for new technologies that enable selective cell typing and detection in complex blood samples, not only between different disease types, but also among healthy blood cells for diagnostic and biosensing purposes.

### 1.3. Challenges in Blood Cell Targeting

Precise molecular targeting of blood cells underpins many of the most impactful modern hematologic technologies. For example, chimeric antigen receptor (CAR)-T cell therapy relies on accurate antigen selection to achieve potent anti-tumor activity, while diagnostic workflows depend on selective isolation of rare cell types from complex blood samples to improve disease detection and classification [26,27]. Beyond clinical settings, selective recognition of blood cell populations is also used in forensics analysis, further underscoring the broad importance of reliable cell-specific targeting [28]. Across these diverse applications, success depends critically on the ability to selectively recognize specific blood cell populations within highly heterogeneous and dynamic mixtures.

Despite the need for and importance of targeting specific cells, achieving selectivity in the blood compartment is inherently difficult. Many cell surface targets are not unique to a single diseased or healthy population, making the choice of an appropriate antigen a central and non-trivial design constraint [29]. For example, CD33, CD117, and CD123, which are upregulated in many AML patients, are also expressed on hematopoietic stem and progenitor cells, creating a substantial risk of off-target engagement without careful tuning of the targeting agent [30,31,32,33,34]. Even when a clinically validated receptor is available (for instance, CD19 for many lymphomas), the development of effective and manufacturable targeting agents against new or less-characterized antigens remains challenging [35]. Together, these factors highlight the need for alternative targeting strategies that can achieve high selectivity in complex blood environments, while minimizing off-target effects and remaining compatible with therapeutic and diagnostic deployment.

### 1.4. Aptamers

The word aptamer, derived from the Latin words aptus (to fit) and meros (part), describes short, single-stranded oligonucleotides that can be composed of a sequence of ribonucleic acid (RNA) or deoxyribonucleic acid (DNA) [36]. These highly versatile RNA or DNA oligonucleotides have the capacity to bind to a specific target through intermolecular interactions. This characteristic has earned them the nickname “chemical antibodies”, reflecting functional analogy rather than structural similarity, while boasting several advantages over traditional antibodies (Table 1). Since their introduction in 1990 by Ellington and Szostak [37] and Tuerk and Gold [38], aptamers have drawn sustained interest for diagnostics, therapeutics and biosensing because their chemical synthesis enables large-scale production, minimal batch variability, low cost, and ease of modification [39,40]. Beyond these advantages, aptamers usually display low immunogenicity, and their activity can rapidly be reversed by a short complementary antidote oligonucleotide, providing on-demand control on in vitro and in vivo function [41].

Highly selective functional nucleic acid aptamers can be generated against a broad spectrum of targets from ions and small molecules to proteins and whole cells [50,51,52,53]. In the field of hematology, numerous aptamers have been selected to recognize surface markers on leukemias and other blood cells, and aptamer–antidote pairs have progressed to clinical trials for coagulation targets illustrating their technical feasibility in blood-related applications [54,55]. Collectively, these properties position aptamers as versatile recognition elements that are well suited for precision therapies and diagnostics.

Several recent reviews have surveyed aptamer applications in hematology, particularly focusing on diagnostics for specific blood cancers, coagulation-related protein targets, or platform-centric biosensing technologies [54,56,57,58,59]. While these studies provide valuable coverage of these domains, they do not address a complementary question, namely how aptamers are being used as targeting reagents for distinct blood cell types, lineages, and functional states. As such, here we present a cell-centric perspective organized around hematopoiesis. Rather than cataloging disease-specific assays or sensing platforms, we focus on aptamers as molecular tools for recognizing and manipulating blood cells across lineages and states, in both healthy and diseased contexts, and use this organization to highlight systematic biases, gaps in coverage, and underexploited opportunities in the current literature.

## 2. Overview and Key Innovations of SELEX

Currently, aptamer discovery relies almost exclusively on in vitro selection strategies called SELEX (Systematic Evolution of Ligands by EXponential enrichment), which can enrich selected aptamers not only for tight binding affinity, but also target specificity, stability, and suitability for downstream applications [60,61,62]. In the context of blood cell targeting, the choice of SELEX methodology is particularly consequential due to the complexity of cell surface architectures, the presence of post-translational modifications, and the challenges associated with validating function in physiologically relevant environments. As a result, adaptations of classical SELEX, including cell-based and in vivo selection strategies, help address the unique constraints of hematologic targets.

### 2.1. Classical, Cell-Based, and In Vivo SELEX Strategies

**Figure 2:**
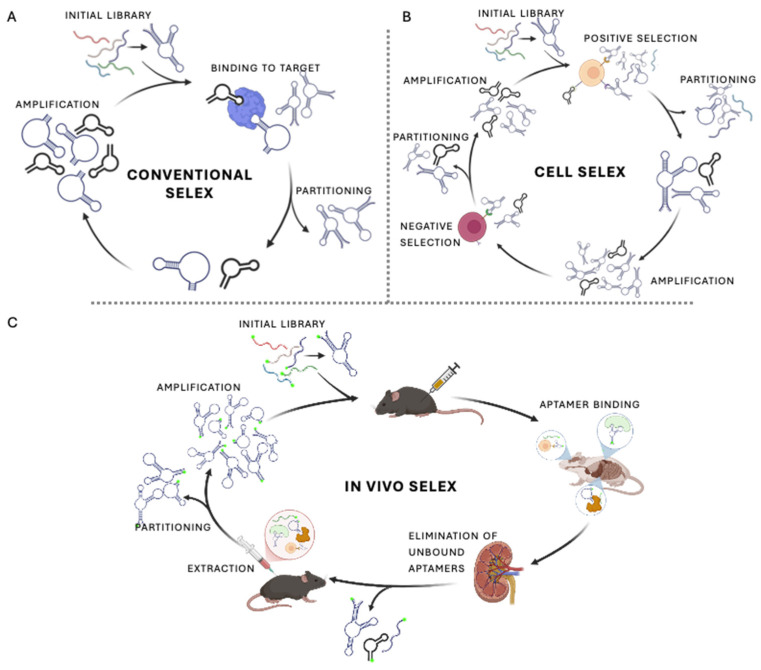
Overview of classical, cell-based, and in vivo SELEX strategies. Schematic comparison of classical SELEX, Cell-SELEX, and in vivo SELEX workflows used for aptamer discovery. (**A**) Classical SELEX is typically performed against purified molecular targets under in vitro conditions, enabling controlled selection but limited representation of native context. (**B**) Cell-SELEX uses intact, live cells to preserve receptor conformation and organization, potentially allowing selection without prior target identification. (**C**) In vivo SELEX introduces aptamer libraries directly into living organisms, enabling selection under physiological conditions that incorporate circulation, tissue access, and biological clearance. The figure highlights how increasing biological complexity across these strategies improves physiological relevance while introducing additional constraints on selection and validation. Created in BioRender. McKeague, M. (2026) https://BioRender.com/tgs0j2h.

In its classical implementation, SELEX is performed against purified molecular targets, with successive rounds of binding, partitioning, amplification, and enrichment used to isolate sequences with measurable affinity and specificity (Figure 2). Although this approach has been widely applied across many target classes, it remains labor-intensive and exhibits a relatively low overall success rate, estimated at approximately 30% [63,64]. These limitations are particularly relevant for blood cell targets, where selections against isolated recombinant proteins often fail to recapitulate the conformational complexity, multimeric organization, and post-translational modifications present on native cell surfaces [61,65,66].

To address these constraints, Cell-SELEX enables selection directly against intact, live cells, thereby preserving membrane context, receptor conformation, and glycosylation patterns [60,61]. Importantly, this strategy removes the requirement for prior target identification and has proven especially valuable in hematologic and malignant systems, where suitable surface markers may be difficult to predict [65,67]. An illustrative example is provided by Daniels et al. (2003) in which aptamers selected against a glioma cell line were subsequently shown to bind tenascin-C following affinity-based target identification [68]. Such studies demonstrate how Cell-SELEX can support both ligand discovery and post hoc identification of relevant molecular targets in complex cellular environments. However, Cell-SELEX, and other in vitro SELEX methodologies, do not fully consider the complex environments in blood, and can lead to poor in vivo functionality.

A related adaptation of Cell-SELEX is ligand-guided selection (LIGS), in which a known high-affinity ligand (e.g., antibody or natural receptor ligand) is used to competitively displace aptamers binding to a specific epitope during selection [69]. This approach enables enrichment of aptamers directed toward predefined cell-surface targets without requiring prior purification of the protein, thereby combining aspects of classical and cell-based SELEX. LIGS can improve target specificity and facilitate epitope-level selection in complex cellular environments, which may be advantageous for hematologic targets with closely related family members [70]. However, LIGS requires the availability of a well-characterized competing ligand and may bias selection toward epitopes overlapping with that ligand, potentially limiting discovery of novel binding sites.

Beyond in vitro approaches, in vivo SELEX introduces aptamer libraries directly into living organisms, allowing selection to occur under physiological conditions that incorporate circulation, tissue access, and biological clearance [71]. Through iterative cycles of administration, recovery, and PCR amplification, aptamers can be enriched against target tissues or circulating cells without prior knowledge of the molecular target. Although this method requires stabilizing modifications (e.g., 2′-fluoro or 2′-O-methyl modifications discussed in Section 2.2.) and must contend with rapid renal clearance, these constraints can enrich for sequences with favorable pharmacokinetic properties [71,72]. Importantly, this approach enables identification of aptamers that retain function in complex biological environments, which partially addresses the gap between in vitro binding and in vivo performance that remains common in blood cell-targeting studies [73]. Despite these notable advantages, in vivo SELEX is rarely used due to its limitations, such as extended selection times, labour intensive, low success rate, and the complexity of utilizing live animal subjects.

### 2.2. Limitations in SELEX for Blood Cell Targeting

The SELEX methodologies (classical, cell, and to a lesser extent in vivo) have been used to generate numerous aptamers across the literature that bind to blood cells [54,74]. However, across these approaches, consistent challenges remain in translating initial binders into robust tools suitable for diagnostic or therapeutic use. In particular, many reported aptamers are characterized primarily under simplified in vitro conditions, with limited evaluation of specificity, stability, or clearance in complex matrices [75]. For example, characterization of aptamers in complex serum or whole blood and clearance properties is often lacking. Furthermore, the reliance on unmodified DNA or 2′-modified RNA without exploring additional options has greatly limited the exploration of backbone diversity.

In practice, chemical modifications are frequently required when aptamers are intended for use in serum, whole blood, or in vivo settings. Unmodified RNA aptamers are particularly susceptible to rapid nuclease degradation, often limiting their functional half-life to minutes in biological fluids [76], whereas DNA aptamers exhibit greater intrinsic stability, but remain vulnerable to endonucleases and rapid renal clearance due to their small size [76,77]. Consequently, a range of chemical strategies have been employed, including 2′-fluoro or 2′-O-methyl substitutions, locked nucleic acid (LNA) incorporation, phosphorothioate (PS) backbone modifications, and molecular weight extension approaches such as PEGylation or albumin-binding conjugates [78,79,80]. Importantly, not all modifications are introduced solely to increase nuclease resistance; some are intended to prolong circulation time, enhance binding affinity, modulate structural rigidity, or reduce immunogenicity. As highlighted in Table 2, the extent and type of modification vary considerably depending on the intended application, and while certain clinical candidates require extensive stabilization, others used in short-term or ex vivo applications may function adequately with minimal modification. Thus, the necessity and degree of chemical modification remain context-dependent rather than universally required.

Moreover, there continues to be challenges in standardization in aptamer literature featuring SELEX, with many past publications lacking detailed information on the sequence, modifications, and validation [75,81,82]. As a result, much of the field remains focused on the identification of aptamer “hits” or using a small selection of existing aptamers, rather than the development of well-characterized “leads”. Future work that incorporates improved validation strategies, expanded chemical diversity, and greater methodological transparency into SELEX workflows could improve the speed and reliability of discovering not only aptamer “hits”, but also moving towards aptamer “leads”.

Implementing in silico designs into SELEX (i.e., machine learning and predictive modeling) may also be a valuable avenue to explore in guiding library design to improve the success rate [83]. However, current computational approaches remain limited. Most structural prediction tools were developed for RNA and do not reliably capture the folding behavior of DNA aptamers, and platforms such as AlphaFold are not yet optimized for predicting aptamer–target interactions. In addition, molecular dynamics simulations are often conducted on nanosecond to microsecond timescales, which may be insufficient to fully sample the conformational flexibility of nucleic acid structures. As such, computational methods should not be viewed as replacements for experimental SELEX. Rather, their current potential value lies in narrowing sequence space, biasing initial library composition, and prioritizing candidates for experimental validation. As larger and more diverse datasets become available, predictive performance may improve, but at present these approaches remain complementary to empirical selection strategies rather than superior to them.

**Table 2:** Examples of reported aptamers targeting blood cell populations: targets, affinities, chemical composition, and sequences. Where applicable, truncated aptamer characterizations were prioritized.

Example Target	^a^ Reported K*_D_*, Measurement Method, and Reference	Relevant Disease	Type	Sequence (5′ → 3′)
A1	29.5 pM [84] ^b^, flow cytometry	N/A ^c^	DNA	CATCCATGGGAATTCGTCGACCCGGGCCTGACAACCTTTACACCTTCTCGAGAAGGTGTCTTCCTAAGCTCGATCTCG
AML1	0.99 ± 0.02 nM [85] ^b^, SPR, truncation	Leukemia	RNA	GGGAUGGACGACCCACCACGGCGAGGUAUCCCAUCCCA
	3.5 ± 0.4 nM [86] ^b^, SPR, truncation	AML	RNA	GGACCCUGCCACGAUAGCGGCGCGGGAAGUAAAGUAUACACCUAACGGUCCA
BAFF-R	47.1 ± 7.6 nM [87] ^b^, gel shift assay	Lymphoma	RNA, 2’fluoro-pyrimidine	GGGAGGACGAUGCGGGAGGCUCAACAAUGAUAGAGCCCGCAAUGUUGAUAGUUGUGCCCAGUCUGCAGACGACUCGCCCGA
BCMA	79.4 nM [88] ^b^, qRT-PCR, truncation	MM	RNA, 2’fluoro-pyrimidine	AGUGCAAGACGUUCGCAGAUUAGCGAAAAGAGGGUCUCAUUGACUAGUAC
CCR5	31.3 nM, [89] ^b^, gel shift assay, truncation	HIV	RNA, 2’fluoro-pyrimidine	GGGAGACAAGACUAGACGCUCAAUGUGGGCCACGCCCGAUUUUACGCUUUUACCCGCACGCGAUUGGUUUGUUUCCC
	110 nM [90] ^b^, flow cytometry	AML	RNA, 2’fluoro-pyrimidine	GGGAGGACGATGCGGGCCUUCGUUUGUUUCGUCCAAGACGACTCGCCCGA
CD3ε	3 nM [91] ^b^, flow cytometry, truncation	N/A ^c^	DNA	GCCGCGGGGTGGGTCTAGTGTGGATGTTTAGGGGGCGGC
	0.3 nM [79], flow cytometry, dimer	N/A ^c^	DNA, LNA, 2′-OMe, PEG-linker	‘+A+GCCGCGGGGTGGGTCTAGTGTGGATGTTTAGGGGGCGGCmUmT- (C42H61N2O10P)4- +A+GCCGCGGGGTGGGTCTAGTGTGGATGTTTAGGGGGCGGCmUmT
	135 nM [92], flow cytometry	N/A ^c^	DNA, PEG-linker	TGGGCGGGGTGGGTCTAGTGTGGATGTTTAGGGGGCCCA
CD3 (murine)	37.9 nM [93] ^b^, SPR	Blood cancer ^d^	RNA, 2’fluoro-pyrimidine	CCTTGCCTGCTTTCACGTGTGATCCCTGCCCGT
CD4	N/A ^c^ [94]	HIV	DNA	GTGACGTCCTGATCGATTGTGCATTCGGTGTGACGATCT
	1.59 nM [95] ^b^, flow cytometry	HIV	DNA	ATCCAGAGTGACGCAGCACCACCACCGTACAATTCGCTTTCTTTTTTCATTACCTACTCTGGC
	2.9 nM [96] ^b^, qPCR	N/A^c^	DNA	GCCTGTTGTGAGCCTCCTAACGATGTCGACGTGCAGCTTCCTTGAGCCTTACTGAAAATACTACCCAGTCCATGCTTATTCTTGTCTCCC
CD8	10.59 nM [97] ^b^, flow cytometry	Stevens–Johnson syndrome	DNA	ACGCTCGGATGCCACTACAGCTTGCTATGCTCCCCTTGGGGTACGTAACGATGTCGACGACTCATGGACGTGCTGGTGAC
	1.9 nM [14] ^b^, flow cytometry	Blood cancer ^d^	DNA	ATCCAGAGTGACGCAGCAACAGAGGTGTAGAAGTACACGTGAACAAGCTTGAAATTGTCTCTGACAGAGGACTAAGCCACCGTGTCCA
CD16α	45 ± 28 nM [98] ^b^, dot blot, truncation	Blood cancer ^d^	DNA, 15-deoxyadenosine linker, PEG	CACTGCGGGGGTCTATACGTGAGGAAGAAGTGGGCAGGTC
CD19	85.4 nM [99] ^b^, flow cytometry	Lymphoma	DNA	TGCGTGTGTAGTGTGTCTGTTCTCCTTTTTTTGGTTGCTGCTCTTAGGGATTTGGGCGG
	N/A ^c^ [100] ^b^	ALL	DNA, RNA	RNA: UUGAAACUGUAAGGUGGC DNA: TTGAAACTGTAAGGTGGC
	49.9 ± 13 nM [101] ^b^, BLI, truncation	B-cell neoplasms	RNA, 2’fluoro-pyrimidine	UGAGCCCUGUUCGACAGGAGGCUCA
CD20	96.91 nM [102] ^b^, flow cytometry	Lymphoma	DNA	ATACCAGCTTATTCAATTGGAATAAGGGGGTATTACTGTCTGGTAAACAAACGCTATGCGAGGGGATTCAAGATAGTAAGTGCAATCT
CD22	N/A ^c^ [103] ^b^	B-ALL	RNA, 2’fluoro-pyrimidine	GGGAGGACGAUGCGGGCCAUUCGUCUUUUCGUCCCCAGACGACUCGCCCGA
CD28 (murine)	40 nM [104] ^b^, filter binding assay, dimer	N/A ^c^	RNA, 2’fluoro-pyrimidine	CAGAGACTTCCAAAATAAAAGACTCCTGAAAGTTGCAAAATAAAAAACTC
CD30	0.11 Nm ^e^ [105] ^b^, SPR	Blood cancers ^d^	RNA, 2’fluoro-pyrimidine	N/A^c^
	50 nM [106] ^b^, flow cytometry	Lymphoma	DNA	TACCAGTGCGATGCTCAG ACTGGGCGAAACAAGTCTATTGACTATGAGCCTGACGCATTCGGTTGAC
CD33	43 nM [107] ^b^, flow cytometry	AML	DNA	TACCAGTGCGATGCTCAGCACGCTTATAGGGGCTGGACAAAATTCTACCCAGCCTTTTCTGACGCATTCGGTTGAC
CD33/CD34	N/A ^c^ [108]	AML	DNA	N/a ^c^
CD38	50.03 nM [109] ^b^, flow cytometry, truncation	MM	DNA	TCCAGAGTGACGCAGCAGCCAACGTGCTTTCTACCTTATTTTCCGTCACTCTCACTCTGGA
	4.8 ± 0.2 nM [110] ^b^, SPR, truncation	MM	DNA	AGGCGCCCAACCTCCTTTAGTGTCAAGGCAGGGGAAACAAGTCTGGCTTAGGGTGT
CD44	187.0 ± 30.6 nM [111] ^b^, filter binding assay	Blood cancer ^d^	DNA, PS	GAGATTCATCACGCGCATAGTCTTGGGACGGTGTTAAACGAAAGGGGACGACCGACTATGCGATGATGTCTTC
	81.3 nM [78] ^b^, fluorescence	Blood cancer ^d^	RNA, 2’fluoro-pyrimidine, 2′-OMe	GGGAUGGAUCCAAGCUUACUGGCAUCUGGAUUUGCGCGUGCCAGAAUAAAGAGUAUAACGUGUGAAUGGGAAGCUUCGAUAGGAAUUCGG
	238 ± 9 nM [112] ^b^, MST, truncation	Blood cancer ^d^	DNA	CATGCTTCCCCAGGGAGATGACCGGGCGTACACCGTCGCGGCACATGTCTGAATGCGTTTAGTCTCTGTG
	14.54 nM [113] ^b^, flow cytometry, truncation	Blood cancer ^d^	DNA	GGGACGCTGAACACTATCATGGGGTGCTATCTCTCTTGGT
	55.5 ± 13.4 nM [114]	Blood cancer ^d^	DNA, PS	TTAAGATCXGXTAGGGAACCAAGACGACAG
CD71	55.02 ± 0.4 nM [115] ^b^, flow cytometry, truncation	Blood cancer ^d^	DNA	ACTCATAGGGTTAGGGGCTGCTGGCCAGATACTCAGATGGTAGGGTTACTATGAGC
	35.2 ± 23.79 nM [116] ^b^, flow cytometry, toehold	Lymphoma	DNA	GGAGTCACACGCATTAGCGTAAAGGGGGTGTTTGTGCGGTGTGGAGTGCGCGTGCTAATGCTGGAGTGTTTCCCAGGACCC
CD117	7.14 nM [117] ^b^, flow cytometry, truncation	AML	DNA	GGGGCCGGGGCAAGGGGGGGGTACCGTGGTAGGAC
	11.75 ± 1.30 nM [118] ^b^, flow cytometry, truncation	AML	DNA	TCCAGTGACGCAGCATCGAGCGGGGGACCCTATTAGCTGAATGAGATGCAATTACAAGCGTGGACACTGGC
	21.8 nM [119] ^b^, BLI	AML	DNA	N/A^c^
CD123	29.41 nM [52] ^b^, flow cytometry	AML	DNA	TGCGTGTGTACTGTGTCTGGTCCCGTAGCTACTAGCGAACTCCCTGCCTCTTAGGGATTTGGGCGG
	39.1 nM [80], flow cytometry	AML	DNA, PS	TGCGTGTGTACTGTGTCTGGTCCCGTAGCTACTAGCGAACTCCCTGCCTCTTAGGGATTTGGGCGG
CD371	15.5 nM [120] ^b^, SPR	AML	DNA	ATTACCAGGGACCGAAGGCAAAACTATGATCGGTGG
CTLA-4	10 nM [121] ^b^, double-filter binding assay, truncation	Blood cancer ^d^	RNA, 2’fluoro-pyrimidine	CCGACGTGCCGCAACTTCAACCCTGCACAACCAATCCGCC
	11.84 nM [122] ^b^, flow cytometry	Blood cancer ^d^	DNA	TCCCTACGGCGCTAACGATGGTGAAAATGGGCCTAGGGTGGACGGTGCCACCGTGCTACAAC
DC-SIGN	21.73 nM [123] ^b^, LSC	N/A ^c^	DNA	GGCGAAAATTTGTGGATATAGAGGGTTACTCGGAT
M0- and M2-like macrophages	22.81 ± 5.6 nM (M0) and 44.12 ± 8.0 nM (M2) [51] ^b^, flow cytometry	Blood cancer ^d^	DNA	GAAGAGTAGATGAAACGTTTTTTCGCCCGATAAAAGGGACGTGCGTCAGACA
mIgM	N/A ^c^ [124] ^b^	Burkitt’s lymphoma	DNA, 5′-iodo-deoxyuridine, PEG, disulfide bond	ACCGGGAGGATAGTTCGGTGGCTGTTCAGGGTCTCCTCCCGGTG
	359 nM [125], flow cytometry, multimers	Burkitt’s lymphoma	DNA, 5′-iodo-deoxyuridine, LNA, 2′-OMe, PEG	ACCGTGGAGGATAGTTCGGTGGCTGTTCAGGGTCTCCTCCACGGT
	N/A ^c^ [69] ^b^, flow cytometry	Burkitt’s lymphoma	DNA	N/A ^c^
MLL-AF9	37.5 ± 2.5 nM [60] ^b^, flow cytometry	AML	DNA	TAGGGAAGAGAAGGACATATGATCGCACACTATTAGAGTGTACGCATGATACATTGACTAGTACATGACCACTTGA
Nucleated red blood cells (NRBC)	38.30 ± 4.99 nM [126] ^b^, flow cytometry, truncation	Prenatal diagnostics	DNA	GCCAAATACCGGTCTGTCGGTGGGTATTGTGGACACTCTGGC
Nucleolin	N/A ^c^ [127] ^b^	Blood cancer ^d^	DNA	TTTGGTGGTGGTGGTTGTGGTGGTGGTGG
OFA/iLRP	101.25 nM [128] ^b^, flow cytometry	AML	DNA	TGCGTGTGTAGTGTGTCTGTTGTTTGTATTGTTGTCTATCCTCTTAGGGATTTGGGCGG
	N/A ^c^ [129], truncation	AML	DNA	TTGTTTGTATTGTTGTCTATCCTCTTAGGGATT
Red blood cell (RBC)	N/A ^c^ [28] ^b^	Forensics	DNA	ATCCAGAGTGACGCAGCACGGGTTGGGGCTGGTTGTGTGTTGTTTTTTTGGCTGTATGTGGACACGGTGGCTTAGT
RhD	580.5 ± 142.0 nM [130] ^b^, flow cytometry	Hemolytic anemia	DNA	GGCCTGGTCTGTTAGCCGGGTAGCAGCCCCGGCACCTATT
Siglec-5	2.77 nM [131] ^b^, flow cytometry	AML	DNA	GACGCTTACTCAGGTGTGACTCGGTACGCCGCAAGACGAGTTGTGTATAAGCCGGCCGAAGGACGCAGATGAAGTCTC

^a^ Functional validation and biological context (e.g., cell line binding, primary patient samples, in vivo models, or diagnostic assay format) are described in the cited primary reports. ^b^ Original SELEX paper for the aptamer. ^c^ N/A indicates that there was no information on the K_D_, sequence, or disease from the reference. ^d^ Blood cancers include B-ALL, T-ALL, AML, and MM, as well as additional types of leukemia and lymphoma. ^e^ Aptamer was discovered for CD30 while selecting for RANK targeting aptamers.

## 3. Aptamers Targeting Blood Cell Types

Most existing reviews of aptamers in hematology organize the literature around diseases, assay formats, or technological platforms. Here, we instead organize the field explicitly around blood cell types and hematopoietic lineages. This framing reflects the fact that many of the central challenges in blood targeting are biological rather than technological, in particular the difficulty of distinguishing closely related cell types, differentiation stages, and functional states within a highly dynamic system. Accordingly, in this section we survey reported aptamers by lineage (B cells, T cells, NK cells, and red blood cells). We use Table 2 as a reference point, and analyze patterns in target selection, chemical composition, and intended application. This organization makes visible both the uneven distribution of aptamer targets across hematopoiesis and the implicit assumptions that have guided target choice, while highlighting underexplored cell types and functional states that represent opportunities for future development.

### 3.1. B Cells

B cells are central to humoral immunity, differentiating into plasma cells that secrete antibodies and regulate adaptive responses [132]. Their relatively well-defined lineage markers and established clinical relevance have made B cells one of the most frequently targeted blood cell types for aptamer development. Accordingly, most reported B-cell aptamers focus on a small set of surface receptors that are already validated in antibody-based diagnostics and therapies [133,134]. This concentration reflects both the accessibility of these targets and a broader tendency to prioritize diseased or malignant B-cell populations over healthy counterparts.

Among reported targets, the bulk of aptamers have been selected against the CD19 receptor. CD19 is a well-known marker for B-cell lineages, but cannot be used to distinguish between healthy and malignant B cells [101]. To date, major efforts in B-cell aptamer development have concentrated on a small number of clinically established targets rather than expanding into less-characterized surface markers. As summarized in Table 2, CD19-targeting aptamers span both DNA- and RNA-based chemistries, with reported affinities generally in the nanomolar range. Despite this volume of work, relatively few studies interrogate how aptamer sequence, chemistry, or structure relate to binding performance at the molecular level. Danquah et al. sought to address this limitation by combining aptamer selection with computational modeling to examine predicted interactions between an RNA aptamer and CD19 [100]. In that study, modeling was used to rationalize an observed improvement in binding affinity upon conversion of the RNA aptamer to a DNA format. Although there is precedent for successful RNA-to-DNA conversion in select aptamers [77,135], such outcomes are not broadly generalizable and likely depend on preservation of key structural motifs rather than simple backbone substitution. However, the proposed binding mechanism was not directly validated experimentally, underscoring a broader limitation in the field, namely the scarcity of structure–function analyses that link aptamer design choices to binding behavior [136].

Beyond CD19, a smaller set of aptamers has been reported against additional B-cell surface receptors, but this landscape remains narrow and conservative. These targets include B-cell maturation antigen (BCMA), CD20, CD22, B-cell activating factor receptor (BAFF-R), and components of the B-cell receptor (BCR) (see Table 2). Notably, the series of studies targeting the membrane-bound B-cell receptor complex stand out as one of the clearest demonstrations of true cell-SELEX against a native, multi-component immune receptor. Moreover, it provides a rare example of aptamer selection guided by biologically grounded receptor architecture rather than by convenience targets. Compared to CD19, aptamers against these targets are fewer in number, despite their established relevance in both normal B-cell biology and B-cell malignancies. Notably, most reported aptamers targeting these receptors exhibit nanomolar affinities and include minimal chemical modifications, reflecting a conservative design space similar to that observed for leukemia-associated targets more broadly. Currently, much of the work on B-cell aptamers has remained focused on therapeutics for diseased lineages. By contrast, aptamer development targeting healthy B cells remains limited, despite their potential utility in biosensing, immune profiling, and diagnostic applications. As a result, most B-cell aptamers have effectively been evaluated in roles closely analogous to antibody reagents, rather than as probes of more subtle distinctions in B-cell state, differentiation, or function. Expanding aptamer selection toward healthy B-cell populations could therefore enable new tools for monitoring immune status and cell composition, while reducing reliance on disease-specific selection paradigms.

### 3.2. T Cells

T cells are central to adaptive immunity, orchestrating cellular responses through distinct subpopulations. Helper (CD4^+^) T cells engage MHC-II on antigen-presenting cells to recruit and regulate immune effectors, while cytotoxic (CD8^+^) T cells recognize MHC-I and directly kill target cells [137,138]. Because of their central role in immune regulation and their widespread use in immunotherapy, T cells have been a major focus of aptamer development among healthy blood cell populations. Accordingly, reported T-cell aptamers span a broader range of intended applications than those developed for B cells, including cell capture, activation, and targeted delivery (see Table 2).

The CD3 receptor, expressed on all T-cell subsets, is a core component of the T-cell receptor (TCR) complex and has been widely studied in SELEX experiments [139]. However, much of the existing literature has focused on improving binding performance or chemical stability of CD3 aptamers rather than demonstrating functional utility in biologically relevant settings [79]. Co-stimulatory activation of CD3 and CD28 receptors leads to T cell activation, and a paper by Pastor et al. explored this by designing an anti-CD28 aptamer and found successful activation of T cells [104]. In that study, however, CD3 engagement relied on an antibody reagent, and the CD28 aptamer was specific to murine targets, limiting direct translational relevance. This highlights a recurring pattern in the T-cell aptamer literature, in which proof-of-concept activity is demonstrated, but integration into fully aptamer-based or human-relevant systems remains incomplete.

Beyond CD3, most T-cell aptamer work has focused on lineage-defining receptors such as CD4 and CD8, a strategy that prioritizes cell-type identification over functional state resolution (see Table 2). Unlike B cells, much of the literature has produced aptamers for healthy T cells, and the intended applications have ranged from drug delivery to specific cell capture. Nevertheless, most of these aptamers do not discriminate between functional T-cell states, such as resting, activated, exhausted, or memory phenotypes, nor do they distinguish closely related subpopulations within the CD4^+^ or CD8^+^ compartments. Hence, most reported T-cell aptamers distinguish cell types more readily than they distinguish cell states. As a result, aptamer-based tools for dynamic immune monitoring remain underdeveloped. Future work in this area could help to improve in situ biosensing, immune profiling, and diagnostic applications, particularly in contexts requiring longitudinal monitoring of patient immune status, and take advantage of aptamers’ desirable characteristics, including low immunogenicity, reversibility, and chemical tunability.

### 3.3. Natural Killer (NK) Cells

NK cells are innate lymphoid cells comprising roughly 2% of circulating lymphocytes and function in antiviral and antitumor immunity via cytokine release and cytotoxicity [140,141]. Unlike T cells, NK cells lack CD3, and their effector activity is determined primarily by CD16 and CD56 expression [142]. Differences in CD16 and CD56 expression define functionally distinct NK-cell subsets with high-CD56 and low-CD16 cells mediating cytokine secretion, but not cytotoxicity. Meanwhile, low-CD56 and high-CD16 cells, which represent approximately 90% of circulating NK cells, mediate both cytotoxicity and cytokine release. Despite growing interest in NK cells for immunotherapy, including CAR-NK strategies, aptamer development targeting NK-cell receptors remains extremely limited.

As summarized in Table 2, only a small number of aptamers have been reported for NK-cell-associated targets, including a single aptamer against CD16 and none directed toward CD56 as of writing [98]. This limited coverage contrasts sharply with the clinical importance of NK cells and reflects a broader underrepresentation of innate immune lineages in aptamer discovery efforts. An illustrative example of functional adaptation is provided by an anti-CD30 aptamer initially selected by Parekh et al. and later repurposed by Yang et al. for NK-cell-mediated targeting [106,143]. In this approach, the aptamer was displayed on the surface of NK cells to direct cytotoxic activity toward CD30-positive cancer cells, functioning as a modular alternative to conventional CAR constructs. While challenges remain, including aptamer stability, surface persistence, and in vivo durability, this strategy highlights the potential of aptamers to enable reversible and tunable NK-cell targeting, offering conceptual advantages over permanent genetic modification.

Despite these proof-of-concept demonstrations, aptamer selection against healthy NK cells and NK-cell-specific functional states remains largely unexplored. This appears to reflect not a lack of biological relevance, but rather potentially a tendency to prioritize targets that are already embedded in established therapeutic and diagnostic pipelines. Expanding aptamer discovery toward NK-cell subsets and activation states could enable new diagnostic, biosensing, and therapeutic strategies, particularly in settings where reversible or transient targeting is desirable. The scarcity of NK-cell aptamers therefore represents both a limitation of the current literature and a clear opportunity for future development.

### 3.4. Red Blood Cells (RBCs)

RBCs are responsible for oxygen transport and play roles in a variety of diseases, including malaria, sickle cell anemia, and sepsis [16,144,145,146]. Unlike lymphoid cells, RBCs lack nuclei and have limited surface protein diversity, which constrains the number of accessible molecular targets for selective recognition. As a result, aptamer development targeting RBCs has been far more limited than for immune cell lineages and has focused primarily on therapeutic modulation and diagnostic applications rather than immune regulation.

As summarized in Table 2, only a small number of publications report aptamers that directly target RBC-associated antigens. Most of these efforts have centered on clinically established blood group antigens, including the A and B antigens and the Rhesus (Rh) factor, which play central roles in transfusion compatibility. In particular, DNA aptamers have been developed to bind the RhD antigen and sterically mask RhD epitopes on RhD^+^ RBCs, thereby reducing recognition by alloanti-RhD antibodies [130]. This strategy illustrates a distinct advantage of aptamers over antibody-based approaches, namely the ability to reversibly modulate immune recognition without permanent alteration of the cell surface.

Beyond blood group antigens, aptamer selection against RBCs has also been explored for forensic identification and prenatal diagnostics, including targeting nucleated red blood cells (NRBCs) (Table 2). Overall, however, target selection in this area appears to have been shaped primarily by practical considerations related to transfusion compatibility and cell identity, rather than by broader exploration of functional or pathological surface features. This focus is understandable given the limited surface proteome of mature RBCs, but it has also meant that aptamer development remains concentrated on a small number of canonical antigens. Expanding aptamer discovery toward additional RBC-associated targets or functional states could enable new diagnostic and transfusion-related applications, particularly where temporary and reversible modulation of RBC recognition is desirable.

### 3.5. Integrative Perspective

Collectively, publications across B cells, T cells, NK cells, and RBCs illustrate the conceptual versatility of aptamers across diverse hematologic contexts, spanning immune modulation, diagnostics, biosensing, and therapeutic targeting. However, analysis of reported aptamers reveals clear and recurring biases in cell type selection and application focus. As summarized in Table 2, the majority of aptamers have been developed against diseased or malignant blood cell populations, with comparatively fewer efforts directed toward healthy cells or physiologically relevant cell states. While many of the targets listed can also be present on healthy cells (e.g., CD3 or CD19), existing aptamers are often optimized under disease-specific or non-physiological conditions, making adaptation to alternative contexts challenging [75]. This limitation is compounded by a general lack of structure–function insight across the aptamer literature. There are currently only a handful of studies that systematically examine how sequence, chemistry, or folding relate to target engagement under biologically relevant conditions [147,148]. As a result, repurposing existing aptamers frequently requires compromise in binding performance or experimental design, rather than rational optimization. Greater integration of structural characterization and functional validation could reduce reliance on repeated, labor-intensive SELEX campaigns, particularly for applications involving healthy blood cells where target epitopes are conserved rather than mutated.

Furthermore, aptamer development has remained heavily skewed toward lymphoid lineages, particularly B and T cells, while innate and non-immune blood cell populations remain underrepresented. Although some studies have explored myeloid targets, these efforts have largely focused on diseased cells, leaving healthy myeloid populations, NK-cell subsets, and RBC-associated targets comparatively unexplored (Table 2). Given the favourable properties of aptamers for reversible binding, low immunogenicity, and chemical tunability, expanding aptamer discovery toward healthy blood cells and functional cell states represents a significant opportunity. Such efforts could enable new tools for immune monitoring, diagnostics, and biosensing that leverage the dynamic and heterogeneous nature of blood rather than treating it solely as a disease target.

## 4. Applications

The application landscape for blood cell-targeting aptamers reflects both the conceptual strengths of aptamers and the current limitations in their development. In principle, aptamers offer capabilities that are difficult to reproduce with antibodies, including reversible binding via antidotes, programmable multivalency, and precise chemical control over structure and function. In practice, however, most reported applications use aptamers as direct substitutes for antibodies in established workflows, rather than exploiting these distinctive features. As a result, the literature is rich in proof-of-concept demonstrations, but relatively limited in studies that interrogate robustness, physiological performance, or translational viability. In the sections below, we organize applications by use case and discuss, where relevant, the extent to which current implementations exploit or underutilize the distinctive properties of aptamers.

### 4.1. Selective Isolation of Blood Cells

**Figure 3:**
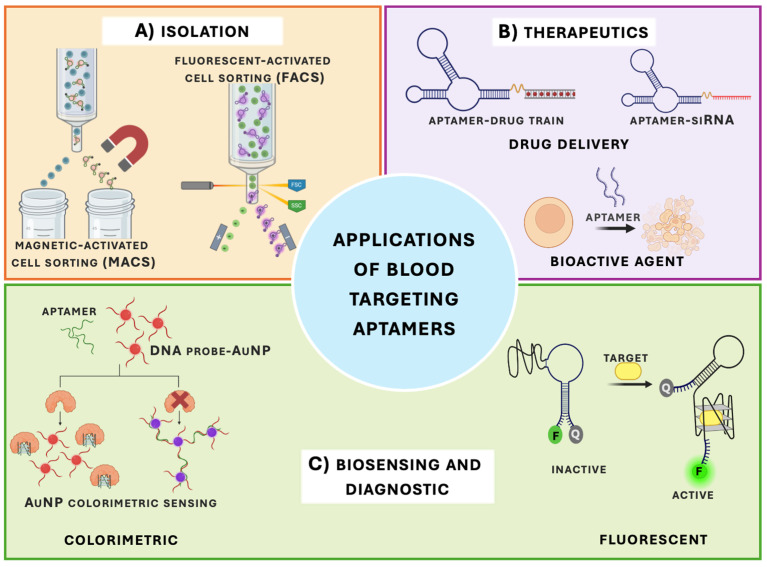
Overview of major applications of aptamers in blood cell research and medicine. This schematic summarizes the principal applications of blood cell-targeting aptamers discussed in this review. (**A**) Aptamers have been successfully integrated into cell isolation platforms such as fluorescence-activated cell sorting (FACS) and magnetic-activated cell sorting (MACS) to achieve high-purity blood cell populations for downstream analyses. (**B**) In addition, aptamers function as therapeutic agents and targeted drug delivery vehicles by selectively binding disease-associated cell surface markers. (**C**) Aptamer-based biosensors (aptasensors) have also demonstrated high sensitivity and versatility across colorimetric and fluorescence-based detection strategies, enabling the detection and monitoring of a wide range of blood-derived analytes. Created in BioRender. McKeague, M. (2026) https://BioRender.com/t2inrn0.

Isolating specific blood cell populations from mixed samples such as peripheral blood or bone marrow is essential for downstream applications in both clinical and research settings (Figure 3). These techniques are essential for reliable biosensing, diagnostics, and even therapeutics (i.e., CAR-T cell therapy) and therefore require highly selective and minimally disruptive separation of target cells. Aptamer-based capture methods have emerged as promising tools for blood cell isolation, offering comparable performance to antibody-based systems while providing improved reproducibility, lower cost, and the potential for reversible cell release [26,43,149]. In this section, we outline the dominant blood cell isolation platforms and discuss how aptamer-specific properties can enhance their performance and flexibility.

Fluorescence-activated cell sorting (FACS) and magnetic-activated cell sorting (MACS) are the two primary techniques used to isolate defined blood cell populations [150,151]. FACS enables multiparametric separation using fluorescent probes and can distinguish closely related immune subsets, such as CD4^+^ and CD8^+^ T cells. However, FACS is associated with high equipment costs, specialized technical requirements, and extended processing times, which can limit scalability and clinical translation [152]. By contrast, MACS relies on magnetic micro- or nanoparticles to capture cells via surface markers (e.g., CD3 for T cells or CD19 for B cells). Although limited to surface antigens and typically restricted to isolating one population per run, MACS offers faster and more cost-effective isolation, with greater compatibility with clinical workflows and good manufacturing practice (GMP) environments. Aptamers have been successfully adapted for both FACS- and MACS-based separation of blood cells, achieving yields and purities comparable to antibody-based systems [14,153]. Their chemical synthesis, reduced batch-to-batch variability, and reversible binding properties make them particularly attractive for isolating diverse hematologic lineages, including T cells, NK cells, and progenitor populations.

Aptamer-mediated separation offers distinct advantages over antibodies for isolating blood cells intended for therapeutic use. Unlike antibodies, aptamers can be designed to release bound cells under mild, non-damaging conditions using complementary “antidote” strands [14,154]. This reversible capture-and-release capability directly addresses a key limitation of antibody-based isolation, namely irreversible receptor occupancy and potential downstream functional perturbation. For example, Kacherovsky et al. selected a DNA aptamer for CD8^+^ T-cell isolation using a MACS-based workflow [14]. The authors demonstrated isolation of highly pure and viable CD8^+^ T cells from peripheral blood mononuclear cells (PBMCs). Excitingly, the performance was comparable to antibody-based capture, while enabling efficient cell release through toehold-mediated strand displacement.

Direct comparisons between aptamers and antibodies are relatively uncommon in the literature. Therefore, antibodies remain the gold standard for blood cell isolation, as well as biosensing, diagnostics, and therapeutics. This is not because aptamers are inferior, but because of entrenched protocols, regulatory familiarity, and decades of infrastructure investment [155]. Systematic, application-relevant benchmarking studies, particularly in whole blood and clinically realistic workflows, would substantially strengthen the case for aptamer adoption. Furthermore, reversible aptamer-based isolation remains underexplored. Adapting this strategy to existing aptamers against other blood cell markers (e.g., CD3, CD16, or CD19) represents a clear opportunity to improve the precision, safety, and scalability of blood cell purification for both research and therapeutic applications.

### 4.2. Therapeutic Applications

#### 4.2.1. Drug Delivery

Apart from their classic functions as molecular recognition elements, aptamers have also been applied as programmable delivery vehicles that can interface with several therapeutic platforms, such as nanoparticles, extracellular vesicles, CRISPR liposome complexes, and engineered bispecific or multivalent architectures (Figure 3) [118,156,157,158]. These strategies rely on aptamers’ high affinity and specificity to achieve cell-selective targeting, while their chemical modularity primarily enables conjugation to therapeutic payloads and integration into more complex delivery architectures. This differs from antibody–drug conjugates (ADCs) where there is efficient drug delivery. For instance, a recent paper by Yu et al. used an anti-CD38 antibody to target multiple myeloma, but the downsides of the antibodies (e.g., limited target selection and payload capacity) can hinder real-world therapeutic outcomes [159,160].

**Figure 4:**
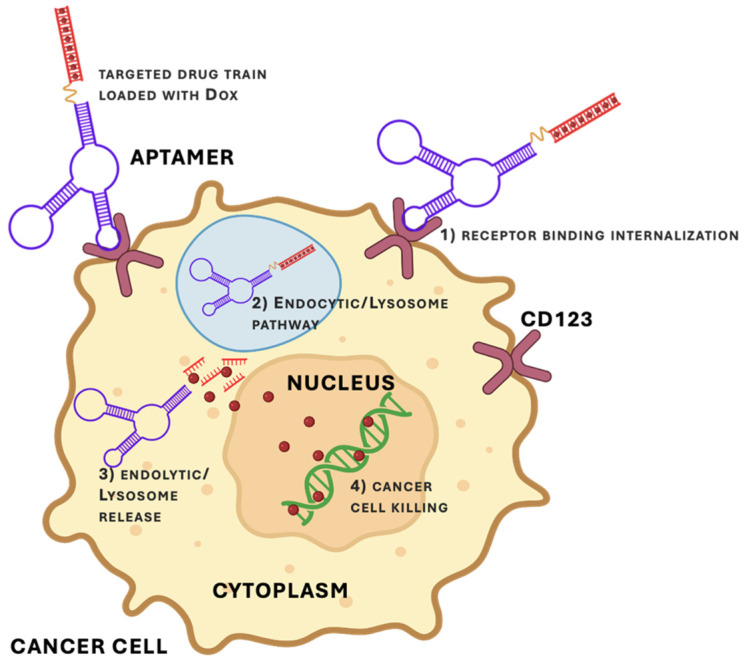
Schematic representation of CD123-targeted aptamer drug delivery. (1) The CD123 aptamer–drug train binds selectively to CD123 on the surface of leukemic cells, enabling receptor-mediated internalization. (2) & (3) Following endocytosis, the aptamer–drug complex is trafficked to endolysosomal compartments, where enzymatic degradation of the DNA scaffold releases the conjugated chemotherapeutic payload. (4) Subsequent nuclear accumulation of the released drug induces cytotoxicity. Figure adapted from the aptamer–drug train design reported by Wu et al. [52]. Created in BioRender. McKeague, M. (2026) https://BioRender.com/t2inrn0.

In the most straightforward implementations, aptamers are directly conjugated to therapeutic cargos, creating single-component targeting constructs in which the aptamer serves simultaneously as the recognition element and the delivery scaffold. For example, for delivery to malignant hematopoietic cells, chemotherapeutic drugs can be directly coupled to an aptamer targeting agent. Take an anti-CD123 aptamer developed by Wu et al., where they utilized a targeted drug chain strategy to link doxorubicin to the ends of their aptamers to treat AML (Figure 4) [52]. Doxorubicin intercalates into GC base pairs, and a GC-rich duplex region was appended to the aptamer to generate a multivalent DNA “drug-train” capable of carrying high drug payloads. In this system, the aptamer serves both as a targeting ligand for CD123^+^ leukemic cells and as a structural scaffold that enhances intracellular drug accumulation.

Aptamer-mediated delivery has also been extended to RNA-based therapeutics in hematologic and immune contexts. A landmark example is the work by Neff et al., who developed an anti-gp120 RNA aptamer fused to an siRNA payload for targeted delivery to HIV-infected CD4^+^ T cells in vivo [161]. In a humanized mouse model with multilineage human hematopoiesis, this aptamer-siRNA chimera selectively entered infected T cells, suppressed viral replication by several orders of magnitude, and protected against CD4^+^ T cell depletion. Notably, the aptamer itself contributed antiviral activity while simultaneously serving as a cell-specific delivery vehicle for the siRNA, illustrating the dual functional potential of aptamer-based targeting constructs in blood and immune cell populations [161,162].

More broadly, strategies based on direct conjugation of aptamers to small-molecule or nucleic acid payloads share limitations with traditional drug-delivery systems, including limited drug stability, poor control over intracellular release, and inefficient endosomal escape [163]. To address these challenges, several groups have incorporated aptamers as targeting ligands on liposomal or polymeric nanoparticles. For example, de la Puente et al. developed an anti-CD38 RNA aptamer to functionalize bortezomib-loaded nanoparticles for MM [164]. Aptamer functionalization significantly enhanced selective nanoparticle accumulation in CD38^+^ myeloma cells, improving therapeutic efficacy in preclinical models. This work illustrates how aptamers can function as modular targeting elements within established nanomedicine frameworks rather than as standalone delivery agents.

#### 4.2.2. Aptamers as Therapeutics

Beyond drug delivery, aptamers themselves have been explored as active therapeutic agents, including in hematopoietic malignancies. A defining and largely unique advantage of aptamer therapeutics is the ability to externally and rapidly control their activity using the complementary “antidote” oligonucleotides described earlier, enabling reversible, titratable intervention in vivo. This property fundamentally distinguishes aptamers from antibodies and small molecules, whose effects cannot be switched off once administered [165,166]. In parallel, the ability of SELEX to identify high-affinity ligands without prior target engineering provides advantages over protein and small-molecule discovery in terms of cost, speed, and chemical control [167]. These features, together with long-term storage stability, scalable synthesis, and the capacity to modulate biological activity through relatively simple tertiary structures, make aptamers especially well-suited for applications where safety, controllability, and temporal precision are critical.

**Figure 5:**
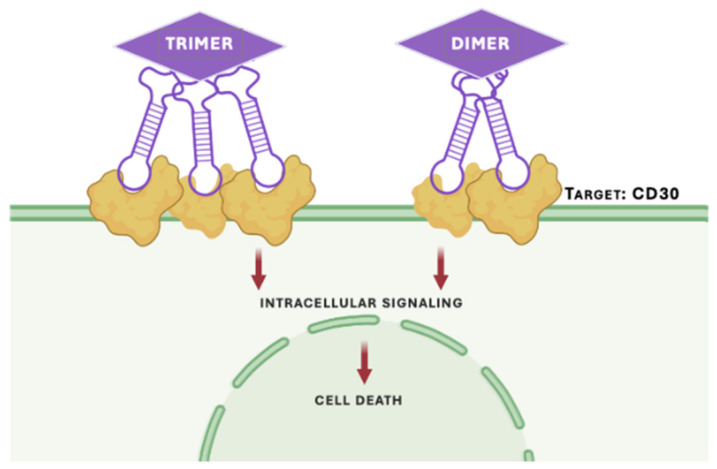
Schematic illustration of multimeric CD30-targeting aptamers for receptor activation. Trimeric and dimeric CD30-specific aptamer constructs bind and cluster CD30 receptors on the surface of target cells, promoting receptor oligomerization and activation of downstream signaling pathways that culminate in apoptosis. Figure adapted from the multimeric therapeutic aptamer design reported by Parekh et al. [106]. Created in BioRender. McKeague, M. (2026) https://BioRender.com/t2inrn0.

While many aptamer-based blood therapies are focused on delivery applications, aptamers can also function as standalone therapeutic agents that directly modulate cell-surface signaling pathways, analogous to antibodies or receptor-targeting biologics. For example, Parekh et al. developed an anti-CD30 aptamer that exploited the pro-apoptotic signaling associated with CD30 engagement in anaplastic large cell lymphoma (ALCL) (Figure 5) [106]. The aptamer selectively induced apoptosis in CD30^+^ cell lines while sparing CD30^−^ controls, demonstrating target-specific cytotoxicity in vitro [106]. Furthermore, Huang et al. selected a DNA anti-CTLA4 aptamer via cell-SELEX that functioned as an agonist of cytotoxic T-lymphocyte antigen-4 (CTLA4) [122]. Notably, this study included in vivo validation, demonstrating antitumor efficacy, serum stability, and low toxicity, and directly compared aptamer performance with an anti-CTLA4 monoclonal antibody. Such comparisons remain rare in the aptamer literature and represent an important benchmark for translational relevance [122].

Beyond oncologic or immune-modulatory applications, aptamers have also been explored as reversible functional blockers in non-malignant blood cell contexts, a niche that particularly highlights their low immunogenicity and programmability. For example, Zhang et al. reported an ssDNA anti-RhD aptamer designed to sterically block RhD antigen recognition to reduce hemolytic anemia risk during transfusion in RhD^−^ patients when compatible blood is scarce [130]. In vitro studies using mixed peripheral blood mononuclear cell populations demonstrated effective masking of RhD epitopes at optimized concentrations. Although in vivo validation is required, this work highlights a therapeutic niche uniquely suited to aptamers, namely the temporary and reversible modulation of immune recognition on healthy blood cells without permanently altering cell function.

Finally, though this review is primarily focused on cellular targets in hematology, the most mature and clinically advanced applications of aptamer therapeutics in the blood have, in fact, been directed against soluble coagulation proteins. Coagulation represents one of the clearest success stories for aptamer therapeutics, because aptamers can bind large functional protein surfaces (often exosites) to block enzyme or cofactor function and, uniquely, can be rapidly shut off via complementary antidotes, an especially valuable property in settings where bleeding risk must be tightly controlled. Although clinical development of the most advanced early system was ultimately halted due to rare but severe adverse reactions associated with PEG, the factor IXa inhibitory aptamer–antidote platform nevertheless demonstrated the core clinical feasibility of this approach [168,169]. In this work, a PEGylated FIXa aptamer paired with an oligonucleotide antidote (the REG1 system) advanced through multiple clinical studies as a rapidly reversible procedural anticoagulant [170]. The phase III REGULATE-PCI trial confirmed potent anticoagulant activity and rapid reversal by the complementary antidote in patients. However, the study was terminated due to hypersensitivity reactions later attributed to pre-existing anti-PEG antibodies rather than failure of the aptamer mechanism itself [170,171]. This experience provided several critical lessons for the field: PEGylation, while extending circulation half-life, is not immunologically inert; aptamer pharmacokinetics must be engineered with careful attention to formulation-associated immune responses; and programmable antidote-mediated control of coagulation is clinically achievable in humans. More recently, Sullenger and coworkers introduced an “EXACT” (Exosite–ACTive site) anticoagulant in which a thrombin exosite-binding aptamer was chemically conjugated to dabigatran, a small-molecule active-site inhibitor [172]. This bivalent construct increased thrombin affinity and inhibitory potency by over three orders of magnitude relative to the aptamer alone and achieved heparin-like anticoagulant activity in vitro, in vivo, and ex vivo, while retaining the programmability and controllability of an aptamer-based system.

### 4.3. Sensing in Diagnostics and Forensics

Aptamer-based biosensors (“aptasensors”) have emerged as powerful tools for detecting and characterizing blood cells and their surface antigens [54,173,174]. By transducing aptamer–target binding events into optical or electrical signals, these systems enable quantitative and often label-free detection of specific blood cell types, in some cases with sensitivities reaching the femtomolar range [175]. Compared to antibody-based assays, aptasensors benefit from the intrinsic properties of aptamers, including high-affinity and specific target recognition, low immunogenicity, low production cost, high chemical stability, and minimal batch-to-batch variability, which together enable faster response times and improved robustness in blood analysis workflows [176,177].

The chemical modularity of aptamers further allows their straightforward integration into diverse sensing formats, including colorimetric and fluorescence-based platforms, making them attractive for both laboratory diagnostics and point-of-care applications. For instance, Costanzo et al. (2024) demonstrated the potential of using aptamers in forensic science to selectively bind and differentiate between RBCs from different individuals [28]. Although currently limited to a single proof-of-concept study, this work suggests potential future applications of aptamers in identity verification and sample authentication using blood-based evidence. Nevertheless, despite their conceptual and technical advantages, a substantial gap remains between aptamer discovery by SELEX and successful deployment in clinically robust biosensing systems [149]. In this section, we highlight representative examples of aptamer-based biosensors for blood cell detection and discuss both their technical promise and their current translational limitations.

A complementary diagnostic use case has also emerged in affinity proteomics, where chemically modified aptamers (“SOMAmers”) enabled high-plex profiling of soluble blood proteins. Shubin et al. (2019) used the SOMAscan platform to quantify 1305 serum proteins from small-volume longitudinal samples and reported a dataset designed to support rejection biomarker discovery in face transplantation [178]. This study provided one of the most detailed end-to-end examples of aptamer-enabled blood profiling, including sample handling constraints (for example, hemolysis and freeze–thaw control) and an openly deposited dataset for downstream analysis. Such platform-scale studies highlighted a second route to blood “diagnostics”, biomarker discovery from serum proteomes, which complemented cell-surface aptasensors and cell-targeting workflows discussed below.

#### 4.3.1. Colorimetric Aptasensors

**Figure 6:**
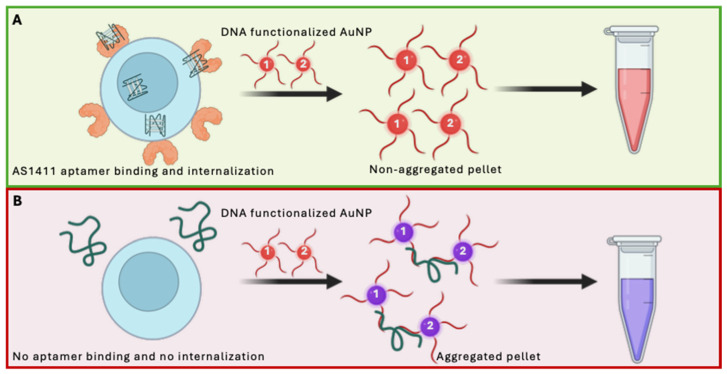
Schematic illustration of colorimetric aptamer-based detection of cell-surface nucleolin. The assay employs the nucleolin-binding aptamer AS1411 and gold nanoparticles (AuNPs) functionalized with complementary single-stranded DNA probes. (**A**) In the presence of nucleolin-expressing cells, AS1411 binds to cell-surface nucleolin and is internalized, preventing AuNP crosslinking and maintaining a dispersed nanoparticle state with a red solution color. (**B**) In the absence of the target receptor, free AS1411 remains in solution and mediates AuNP aggregation through probe hybridization, resulting in a visible color change to purple. Figure adapted from the colorimetric cancer cell detection strategy reported by Borghei et al. [179]. Created in BioRender. McKeague, M. (2026) https://BioRender.com/t2inrn0.

Colorimetric aptasensors provide a simple and cost-effective route to visual detection of blood cell biomarkers [179,180]. A common implementation uses gold nanoparticles whose aggregation state, and thus optical properties, change upon aptamer–target binding, producing a visible color shift. Two commonly used colorimetric models are the adsorption–desorption approach and the hybridization approach. In the adsorption–desorption model the aptamer is non-specifically adsorbed to the surface of the AuNP and desorbs in the presence of its target [181]. This model makes several assumptions: (1) the aptamer has a higher affinity for its target than the surface of the AuNP, (2) the aptamer detaches from the surface of the AuNP, and (3) the colour shift that occurs is from the unprotected AuNPs in the presence of salt. Researchers such as Liu et al. and Chen et al. have challenged of these assumptions and chemical oversimplifications and shown that this model is sometimes prone to artifacts [182,183]. The hybridization model, however, has emerged as a robust option that utilizes predictable interactions. One representative example is the nucleolin-targeting AS1411-based system reported by Borghei et al. in which the aptamer was hybridized to DNA probes immobilized on gold nanoparticles for cancer cell detection (Figure 6) [179]. The aptasensor showed high specificity and high sensitivity for cancer cells. However, like many colorimetric aptasensors, its performance has not yet been demonstrated in whole blood, where matrix complexity and the strong optical background from hemoglobin pose significant challenges. Despite these limitations, the conceptual simplicity of this approach makes colorimetric aptasensors attractive for rapid screening applications in low-resource or field settings [149].

#### 4.3.2. Fluorescent Aptasensors

**Figure 7:**
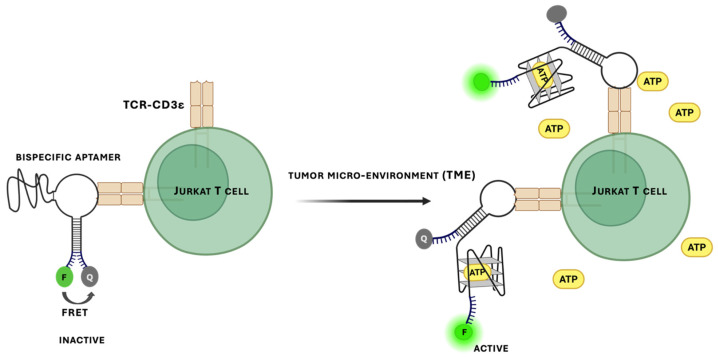
Tumor microenvironment-responsive bispecific fluorescent aptamer sensor. The bispecific aptamer is designed to simultaneously recognize the TCR–CD3ε complex on Jurkat T cells and extracellular ATP, a metabolite present at 10^3^–10^4^-fold higher concentrations within the tumor microenvironment. In the absence of ATP, the construct adopts an inactive conformation in which the Förster resonance energy transfer (FRET) donor–acceptor pair remains in proximity, resulting in quenched fluorescence. Elevated ATP levels induce a conformational transition to the active state, spatially separating the FRET pair and producing a fluorescence signal. This design enables conditional activation of cell-surface sensing based on metabolic cues, improving specificity for malignant environments. Figure adapted from the FRET-based aptamer sensor described by Boykoff et al. [92]. Created in BioRender. McKeague, M. (2026) https://BioRender.com/t2inrn0.

Fluorescent aptasensors are among the most widely used formats for blood cell detection and imaging due to their high sensitivity and compatibility with spatially resolved readouts [184]. One common design, molecular beacon-type aptasensors, employ fluorophore–quencher pairs attached to aptamers such that target binding induces a conformational change and restores fluorescence. Label-free approaches employing intercalating dyes or nanomaterials have also been reported [176]. Boykoff et al. developed a bispecific fluorescent aptasensor responsive to the tumor microenvironment by linking ATP- and CD3-binding aptamers (Figure 7) [92]. Increased ATP levels triggered fluorescence in Jurkat T-cell models. While this work illustrates the conceptual flexibility of fluorescent aptasensor designs, the system was not evaluated in whole blood or in vivo contexts, limiting conclusions about translational utility.

## 5. Summary, Challenges, and Future Perspectives

Aptamers are versatile molecular tools for blood cell applications spanning selective isolation, drug delivery, biosensing, and therapeutics. Throughout this review, we have highlighted how aptamers can exploit differences in blood cell surface marker expression and cellular state, offering advantages such as chemical synthesis, batch-to-batch consistency, reversibility, and low immunogenicity. These properties position aptamers as attractive alternatives or complements to antibody-based approaches in both diagnostic and therapeutic settings.

Despite this promise, substantial barriers remain to translating blood cell-targeting aptamers from benchtop studies to real-world applications. Across application areas, a recurring limitation is the lack of validation in physiologically relevant environments, particularly whole blood or serum. Aptamer-mediated blood cell isolation illustrates this gap clearly: while reversible capture and release has been demonstrated, relatively few studies systematically evaluate isolation efficiency, target internalization, or functional consequences under clinically realistic conditions. Similar challenges arise in drug delivery, where most aptamer-based systems remain at an early stage, relying on relatively simple conjugation or nanoparticle targeting strategies without fully exploiting the programmable logic and release mechanisms that aptamers can offer. In diagnostics and biosensing, many aptasensor platforms demonstrate excellent performance under simplified conditions, yet lack in vivo or complex-matrix validation, limiting their translational impact [185].

More broadly, the current aptamer literature remains skewed toward a narrow subset of blood cell targets, particularly B and T lymphocytes, with comparatively limited exploration of healthy cell populations, innate immune cells, and dynamic cell states. This focus constrains the development of aptamer-based biosensors and diagnostics intended for immune monitoring or longitudinal profiling. In parallel, the absence of standardized good manufacturing practice (GMP) guidelines and regulatory pathways for aptamers continues to slow clinical translation, particularly when compared with well-established antibody platforms [185,186,187].

Addressing these challenges represents a clear opportunity for the field. Future progress will depend on integrating rigorous validation in complex biological matrices, expanding target selection beyond diseased lymphoid cells, and establishing clearer standards for aptamer characterization, manufacturing, and clinical development. With these advances, aptamers are well positioned to move beyond proof-of-concept studies and realize their potential as practical tools for hematopoietic targeting, diagnostics, and therapy.

## Figures and Tables

**Table 1:** Comparison of key properties of aptamer and monoclonal antibodies.

Attributes	Aptamers	Monoclonal Antibodies
Size	10–30 kDa Small size displaying better internalization [42]	~150–170 kDa Restricted internalization—suboptimal for hematologic targets
Stability [43]	Longer shelf life, stable over a wide temperature range Can be transported at ambient temperature Reversible denaturation	Sensitive to temperature Irreversible denaturation
Modification	Easy and versatile site-specific modification Can be modified at both the 5′ and 3′ end, and internally. Modifications can be done during solid phase synthesis or post synthesis	More complex and difficult to do site-specific modifications
Production time [43]	Several weeks to a few months	Several months
Development process	Sequences are chemically synthesized [39,44] SELEX is rapid and tunable to suit application needs Low batch-to-batch variation Scalable production	In vivo production Cell culture Higher batch-to-batch variation
Long-term shelf life	Aptamer sequences can be stored physically or documented digitally then synthesized at production sites [45]	Frozen cell stocks must be maintained for production
Immunogenicity [43]	Low to no immunogenicity [46,47]	Immunogenic
On-demand control/reversibility	Activity can be rapidly reversed with a complementary antidote strand	Lack of on-demand control once dosed
Pharmacokinetic properties	Prone to nuclease degradation and rapid renal clearance [48] Require stabilization	Large, stable proteins with prolonged circulation and slow renal clearance
Target heterogeneity	Able to distinguish isoforms, post-translational variants and conformational changes [49]	Variants can reduce antibody binding accuracy

## Data Availability

No new data were created or analyzed in this study.

## Abbreviations

The following abbreviations are used in this manuscript:

ALCL	Anaplastic large cell lymphoma
ALL	Acute lymphoblastic leukemia
AML	Acute myeloid leukemia
ATP	Adenosine triphosphate
AuNPs	Gold nanoparticles
BAFF-R	B-cell activating factor receptor
B-ALL	B-cell acute lymphoblastic leukemia
BCMA	B-cell maturation antigen
BCR	B-cell receptor
BLI	Bio-layer interferometry
CAR	Chimeric antigen receptor
CCR5	C-C chemokine receptor type 5
CLL	Chronic lymphocytic leukemia
CML	Chronic myeloid leukemia
CTLA4	Cytotoxic T-lymphocyte antigen-4
DC-SIGN	Dendritic cell-specific intercellular adhesion molecule-3 grabbing non-integrin
DNA	Deoxyribonucleic acid
FACS	Fluorescent-activated cell sorting
FRET	Förster resonance energy transfer
GMP	Good manufacturing practice
HIV	Human immunodeficiency virus
HSC	Hematopoietic stem cell
kDa	Kilodaltons
LNA	Locked nucleic acid
LSC	Liquid scintillation counting
MACS	Magnetic-activated cell sorting
MHC	Major histocompatibility complex
mIgM	Membrane immunoglobulin M
MST	Microscale thermophoresis
MM	Multiple myeloma
NK	Natural killer
NRBC	Nucleated red blood cell
OFA/iLRP	Oncofetal antigen-immature laminin receptor protein
PCR	Polymerase chain reaction
PEG	Polyethylene glycol
PS	Phosphorothioate
qRT-PCR	Quantitative real-time polymerase chain reaction
RBC	Red blood cell
Rh	Rhesus factor
RNA	Ribonucleic acid
SELEX	Systematic evolution of ligands by exponential enrichment
siRNA	Small-interfering RNA
SPR	Surface plasmon resonance
T-ALL	T-cell lymphoblastic leukemia
TCR	T-cell receptor
